# Total Neoadjuvant Therapy Outcomes and Watch-and-Wait Feasibility in Locally Advanced Rectal Cancer: A Single-Institution Retrospective Cohort Study

**DOI:** 10.3390/cancers18081200

**Published:** 2026-04-09

**Authors:** Manuel Ramanović, Franc Anderluh, Ana Jeromen Peressutti, Petar Korošec, Irena Oblak, Ajra Šečerov Ermenc, Vaneja Velenik

**Affiliations:** 1 Department of Radiation Oncology, Institute of Oncology Ljubljana, Zaloška cesta 2, SI-1000 Ljubljana, Sloveniavvelenik@onko-i.si (V.V.); 2 Faculty of Medicine, University of Ljubljana, Kongresni trg 12, SI-1000 Ljubljana, Slovenia

**Keywords:** total neoadjuvant therapy, rectal cancer, neoadjuvant chemotherapy, radiotherapy sequencing, watch-and-wait, organ preservation, pathological complete response, circumferential resection margin, extramural vascular invasion, IMRT-SIB

## Abstract

**Background/Objectives**: Total neoadjuvant therapy (TNT), integrating systemic chemotherapy and radiotherapy before surgery or surveillance, has become a standard approach for locally advanced rectal cancer (LARC). However, optimal sequencing strategies and long-term outcomes of watch-and-wait (W&W) following sandwich TNT remain insufficiently characterized. We evaluated oncologic outcomes and treatment response in patients treated with an institutional sandwich TNT protocol. **Methods**: We conducted a retrospective cohort study of consecutive patients with LARC treated with sandwich TNT (induction chemotherapy followed by hypofractionated intensity-modulated radiotherapy with simultaneous integrated boost [IMRT-SIB] chemoradiotherapy and consolidation chemotherapy) at the Institute of Oncology Ljubljana between 2016 and 2023. The primary endpoint was an overall complete response (CR; pathological [pCR] and clinical [cCR]). Secondary endpoints included tumor regression grade (TRG), major pathological response (MPR), R0 resection rate, organ preservation, overall survival (OS), and disease-free survival (DFS). **Results**: Among 205 patients (median age 61 years), overall CR was 29.5% (pCR 19.3% and cCR 10.2%). Major pathological response (TRG 3–4) occurred in 37.6%. R0 resection was achieved in 94.5%. In the W&W cohort (n = 21), local regrowth occurred in 33.3% (95% CI, 14.6–57.0%) over a median follow-up of 4.96 years. Total mesorectal excision (TME)-free survival at 5 years was 73.1% (95% CI, 55.0–97.2%). Estimated 5-year OS was 81.1% (95% CI, 75.5–87.2%) and 5-year DFS was 75.2% (95% CI, 69.0–82.0). In multivariable analysis, non-R0 resection (HR 6.06, 95% CI, 1.99–18.42), MRI circumferential resection margin positivity (HR 3.11, 95% CI, 1.53–6.33), and MRI extramural vascular invasion positivity (HR 1.97, 95% CI, 1.05–3.91) remained independent predictors of DFS. **Conclusions**: Institutional sandwich TNT yields meaningful tumor response and durable survival in MRI-defined high-risk LARC. Structured W&W offers organ preservation with acceptable oncologic control under intensive surveillance.

## Simple Summary

Advanced rectal cancer is increasingly treated with total neoadjuvant therapy (TNT)—a strategy delivering all chemotherapy and radiation before surgery—to improve survival and enable some patients to avoid surgery through watch-and-wait. We studied 205 patients with high-risk rectal cancer treated with a “sandwich” TNT protocol (chemotherapy before and after radiation) at our cancer center. Complete tumor disappearance occurred in 30% of patients, with excellent 5-year survival (81%). Among patients choosing watch-and-wait after complete response, two-thirds preserved their rectum long-term; though careful monitoring was essential as one-third experienced tumor regrowth requiring delayed surgery. Importantly, MRI-detected high-risk features and surgical margin quality remained the strongest predictors of outcome. These findings support the feasibility of sandwich TNT in real-world practice and suggest watch-and-wait as a viable organ preservation option when combined with rigorous surveillance.

## 1. Introduction

The management of locally advanced rectal cancer (LARC) has undergone continuous evolution since the late 1980s, driven by improvements in surgical technique, radiotherapy, systemic therapy, and imaging [1,2,3,4,5,6]. The introduction of total mesorectal excision (TME) in 1986 marked a pivotal enhancement in local control, reducing local recurrence rates from the prior 30–40% to approximately 5–10% [1]. Building on TME, the Swedish Rectal Cancer Trial (1997) and the Dutch trial (2001) demonstrated that preoperative short-course radiotherapy (SCRT) further reduced local recurrence to 3–11% compared with surgery alone (8–27%) [2,3].

The EORTC 22921 trial (2004) and the FFCD 9203 trial (2006) demonstrated that fluorouracil added to preoperative radiotherapy halved the local recurrence rates (approximately 8% vs. 16%) [4,5]. The CAO-ARO-AIO 04 trial brought additional evidence that preoperative chemoradiotherapy (CRT) reduced local recurrence compared with postoperative CRT (6% vs. 13%, *p* = 0.006), with improved tolerability [6]. However, these advances in locoregional control—while highly effective with modern 5-year local recurrence rates < 5%—left unaddressed the persistent problem of distant metastatic disease, occurring in 25–35% of patients at 5 years [1,6]. Additionally, adjuvant chemotherapy completion remained suboptimal due to postoperative morbidity and reduced functional reserve [1,2].

In recent years, the therapeutic landscape for LARC underwent a substantial paradigm shift with the adoption of total neoadjuvant therapy (TNT) [7,8,9,10]. The pivotal PRODIGE-23 trial (2021–2024) randomized 461 LARC patients to induction TNT with six cycles of mFOLFIRINOX followed by CRT plus surgery and adjuvant chemotherapy versus standard CRT followed by surgery and adjuvant chemotherapy [11,12]. With a 7-year follow-up, the induction TNT arm demonstrated significant gains: a 5.1% improvement in disease-free survival (DFS), a 5.8% improvement in overall survival (OS), and a 6.9% improvement in metastasis-free survival. Concurrently, the RAPIDO trial (2021) demonstrated that short-course radiotherapy followed by chemotherapy before TME reduced disease-related treatment failure and approximately doubled pCR rates compared with conventional CRT [9].

The MERCURY trial (2006) established magnetic resonance imaging (MRI) as the criterion standard for staging, demonstrating 92% specificity for predicting negative circumferential resection margin (CRM) [13,14]. Beyond CRM, MRI-based assessment of extramural vascular invasion (EMVI), tumor deposits, and mesorectal fascia involvement guides treatment intensification [14,15,16]. A contemporary analysis by Lord et al. (2022) demonstrated that MRI-defined high-risk features (positive CRM, TD, or EMVI) predicted prognosis superior to TNM staging alone, underscoring the primacy of radiologic risk factors in tailoring TNT intensity [16].

Emerging evidence has validated organ-preservation strategies via watch-and-wait (W&W) in carefully selected patients after TNT. The OPRA trial demonstrated that selective W&W after clinical complete response (cCR/near-cCR) can achieve substantial rectum preservation rates without apparent detriment to disease-free or overall survival [10,17]. The International Watch-and-Wait Registry, encompassing 880 patients across multiple institutions, reported regrowth rates of approximately 20–25% with careful surveillance protocols; importantly, 71.4–100% of patients with regrowth underwent salvage TME with R0 resection achieved in all [18]. These data establish W&W as a feasible option for organ preservation, particularly relevant for younger patients with longer life expectancies who wish to avoid the long-term morbidity of permanent colostomy or sexual/urinary dysfunction [19].

This retrospective observational cohort study examines TNT outcomes in patients with LARC treated at a national referral center using a sandwich TNT strategy. We characterized TNT delivery, response endpoints (pCR, TRG, and cCR with organ preservation), and survival outcomes to assess real-world feasibility and to contextualize results against published TNT trial benchmarks and W&W registries.

## 2. Materials and Methods

### 2.1. Study Design and Patient Population

**Figure 1:**
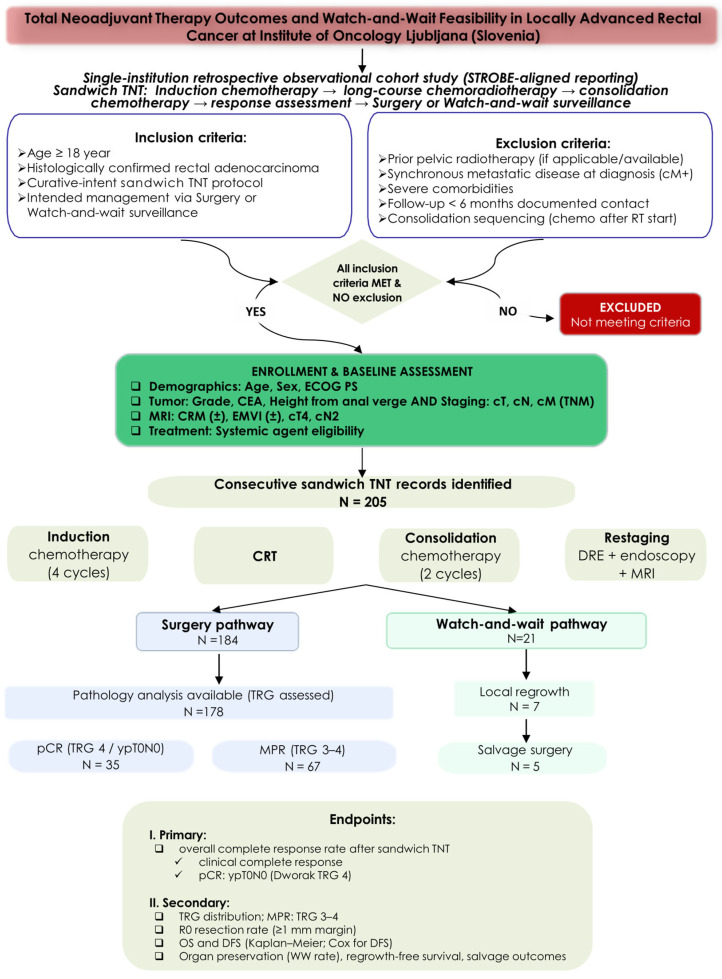
Flowchart of study protocol. CEA, carcinoembryonic antigen; cM/cM+, clinical M stage/clinical M1 (synchronous metastatic disease); cN, clinical nodal stage (N in TNM); CRM, circumferential resection margin; CRT, chemoradiotherapy; cT, clinical tumor stage (T in TNM); DFS, disease-free survival; DRE, digital rectal examination; ECOG PS, Eastern Cooperative Oncology Group performance status; EMVI, extramural venous invasion; MPR, major pathological response; MRI, magnetic resonance imaging; OS, overall survival; pCR, pathological complete response; R0, complete resection with negative margins (≥1 mm margin); TNM, tumor, node, and metastasis classification; TNT, total neoadjuvant therapy; TRG, tumor regression grade; W&W, watch-and-wait; ypT0N0, pathological stage T0N0 after neoadjuvant therapy.

This retrospective observational cohort study included consecutive adult patients with histologically confirmed rectal adenocarcinoma treated with the institutional sandwich TNT protocol at the Institute of Oncology Ljubljana between January 2016 and December 2023 (Figure 1). The study followed STROBE (Strengthening the Reporting of Observational Studies in Epidemiology) recommendations for reporting [20]. The study was conducted in accordance with the Declaration of Helsinki and approved by the institutional ethics committee; informed consent was waived due to retrospective analysis of de-identified data.

### 2.2. Eligibility Criteria

#### 2.2.1. Inclusion Criteria

• Histologically confirmed primary adenocarcinoma of the rectum
• Clinical staging: cT3–4 and/or cN+ disease, cM0 (no distant metastases)
• Performance status: Eastern Cooperative Oncology Group (ECOG) 0–2
• Fitness for TNT: Adequate organ function to tolerate chemotherapy and radiotherapy
• Minimum of 6 months of follow-up available (unless death occurred earlier)

#### 2.2.2. Exclusion Criteria

• Distant metastatic disease (cM1)
• Prior pelvic radiotherapy or systemic therapy for rectal cancer
• Pregnancy or nursing status
• Severe comorbidities precluding neoadjuvant therapy
• Non-evaluable/inadequate quality preoperative imaging
• Substantial missing key baseline staging data (defined as missingness in ≥1 key MRI staging domain or >20% missing among prespecified variables)

### 2.3. Sandwich TNT Protocol and W&W Entry Criteria

Preoperative radiotherapy was delivered using intensity-modulated radiation therapy with a simultaneous integrated boost (IMRT-SIB) according to the institutional protocol previously described by But-Hadzic et al. [21]. Patients underwent CT simulation in the supine position with a full-bladder protocol, with optional MRI fusion for target delineation. Target volumes were defined according to International Commission on Radiation Units and Measurements (ICRU) recommendations, with the gross tumor volume based on MRI and CT findings and clinical target volumes encompassing the mesorectum and regional lymphatic drainage areas [21,22]. Planning target volumes were generated using institutional margins accounting for organ motion and setup uncertainty.

Radiotherapy was delivered using IMRT with inverse planning and daily image guidance. The pelvic planning target volume received 41.8 Gy in 22 fractions, with a simultaneous integrated boost to the primary tumor to 46.2 Gy for cT3 tumors and 48.4 Gy for cT4 tumors. Treatment was administered in five fractions per week. Chemotherapy consisted of the CAPOX regimen: oxaliplatin 130 mg/m^2^ intravenously on day 1 and capecitabine 1000 mg/m^2^ orally twice daily on days 1–14, repeated every 3 weeks. Four cycles were given as induction therapy, followed by IMRT-SIB CRT with concurrent capecitabine (825 mg/m^2^ twice daily) throughout the radiotherapy course, and then two additional cycles of CAPOX as consolidation therapy. Detailed technical aspects of contouring, dose prescription, organ-at-risk constraints, and treatment delivery have been reported previously [21].

Entry into the W&W pathway was restricted to patients with a cCR or carefully selected near-cCR after completion of sandwich TNT. Restaging was based on concordant multidisciplinary reassessment integrating digital rectal examination, endoscopy, and pelvic MRI. Routine repeat biopsy was not required when the combined clinical, endoscopic, and MRI findings were fully consistent with cCR; histologic reassessment was reserved for equivocal or suspicious residual abnormalities [23,24,25]. W&W was considered only when intensive surveillance and timely salvage surgery were both feasible.

### 2.4. Data Collection and Variables

Data were extracted from electronic medical records into a standardized database, including demographics, staging, treatment delivery, pathology, and outcomes. Baseline MRI variables included clinical T/N stage, CRM status, mrEMVI status, and tumor height from the anal verge. CRM involvement was defined as tumor ≤ 1 mm from the mesorectal fascia, consistent with commonly used MRI criteria [26]. mrEMVI was recorded on baseline MRI per radiologic criteria describing tumor signal within extramural vessels beyond the muscularis propria [27].

Radiotherapy-related data were captured as total dose (Gy), fractionation, completion, and interruptions. Chemotherapy variables included regimen, intended and delivered cycles, dose reductions/delays, discontinuation and reason, and relative dose intensity. TNT sequencing was defined by the timing of chemotherapy initiation relative to radiotherapy.

Surgery variables included surgical procedure type and date. Pathology variables included ypT/ypN, resection margin status (R0/R1/R2), lymph node yield, lymphovascular invasion (LVI), perineural invasion (PNI), tumor budding, microsatellite instability (when available), and tumor regression grade (TRG) using the Dworak 0–4 classification [28].

For W&W surveillance, we recorded W&W entry, surveillance assessments, local regrowth events, and salvage surgery details.

### 2.5. Outcome Definitions

#### 2.5.1. Primary Endpoint

The primary endpoint was the overall complete response rate after sandwich TNT, defined as the proportion of patients achieving either a cCR at restaging or a pathological complete response (pCR) after surgery. cCR assessment and W&W entry criteria are described in Section 2.3.

#### 2.5.2. Key Secondary Endpoints

Prespecified survival outcomes were reported at two complementary levels. Overall survival (OS) and disease-free survival (DFS) were estimated for the entire institutional TNT cohort to reflect real-world outcomes of the treatment protocol as a whole. Because allocation to surgery versus W&W was not randomized and depended on response and clinical judgment, any pathway-specific comparisons were considered descriptive only.

Definitions of key secondary endpoints:

1. OS: time from diagnosis to death from any cause, with patients alive censored at last follow-up.
2. DFS: time from diagnosis to first documented disease recurrence (local, regional, or distant) or death from any cause, whichever occurred first, censored at last follow-up.

Additional prespecified outcomes were defined to characterize treatment response, organ preservation, and locoregional control:

1. Major pathological response (MPR): Dworak TRG 3–4 (good or complete regression) [28].
2. R0 resection rate: proportion of resections with all margins negative, defined as a minimum clearance ≥ 1 mm.
3. Organ preservation: W&W enrollment rate among the TNT cohort and durability of rectal preservation over time.
4. W&W regrowth: incidence, timing, and clinical/radiological characteristics of local regrowth in patients managed non-operatively after initial cCR. Local regrowth was defined as tumor reappearance at the primary site after cCR during non-operative management, distinct from post-resection local recurrence. Regrowth-free survival was calculated from entry into W&W protocol to first regrowth or censoring at last follow-up.
5. Salvage surgery outcomes: feasibility of salvage TME for regrowth, R0 resection rate after salvage, and DFS following salvage surgery.
6. Post-resection local recurrence: crude incidence and landmark local pelvic recurrence rates among surgically treated patients. Because the exact date of local recurrence was not consistently documented in all cases, post-resection local control was evaluated using incidence proportions and landmark recurrence rates rather than formal local recurrence-free survival curves.

### 2.6. Statistical Analysis

Continuous variables are presented as median (IQR), and categorical variables as count (%). Baseline characteristics were compared using Wilcoxon rank-sum tests and Fisher exact tests.

pCR rates are presented with an exact 95% CI, using the Clopper–Pearson method. OS, DFS, and regrowth-free survival were estimated using the Kaplan–Meier method with log-rank tests. Survival is presented at 2, 3, and 5 years with 95% CI. Regrowth incidence and time-to-regrowth are presented descriptively. Among patients with regrowth, salvage surgery rates, margins achieved, and subsequent DFS are reported.

Multivariable Cox proportional hazards models were used to identify DFS predictors; model discrimination was assessed using Harrell’s C-index [29]. The proportional hazards assumption was tested using Schoenfeld residuals [30].

All *p*-values are two-sided, and *p* < 0.05 is considered statistically significant. No adjustment for multiple comparisons applied given the exploratory nature. R statistical software (version 4.3.2, R Core Team, R Foundation for Statistical Computing, Vienna, Austria) was utilized for the analysis.

### 2.7. Ethics

The Slovenian National Medical Ethical Committee (decision number: 0120-298/2019/5), Institutional Review Board (10 January 2019) and Institutional Ethical Committee (24 January 2019) approved the study. Due to the retrospective nature of the study, the need to obtain informed consent from participants was waived. All patient identifiers were removed, and analysis was conducted on de-identified data.

## 3. Results

### 3.1. Study Cohort and Baseline Characteristics

A total of 205 consecutive patients with rectal adenocarcinoma received induction-first approach TNT. Baseline characteristics are detailed in Table 1. The median age was 61 years (IQR: 52–67); 65% (n = 133) were male. Most patients presented with cT3–4 disease (98%, n = 200) and cN2 stage (60%, n = 124). CRM was threatened/involved in 61% (n = 122), and EMVI was positive in 66% (n = 120). Tumor grade was well/moderate in 87% and poor/undifferentiated in 13%. Among 205 patients, 184 (90%) underwent surgical resection, and 21 (10%) were managed with W&W surveillance.

Treatment compliance with the planned TNT regimen was robust across the entire cohort. All 205 patients who began induction chemotherapy proceeded to radiotherapy, and all completed the planned IMRT-SIB course, corresponding to a completion rate of 100% in the overall cohort as well as in both the surgically treated and W&W groups.

The majority of patients completed the planned systemic therapy, with 90% of patients in the overall cohort receiving all six planned chemotherapy cycles. Chemotherapy dose reductions were uncommon, and 93% of patients in the overall cohort completed treatment without dose reduction.

Chemotherapy discontinuation occurred in 8.8% of patients overall, including 8.7% in the surgery group and 9.5% in the watch-and-wait group. Discontinuation was most frequently related to treatment-related toxicity, and no treatment discontinuations occurred due to patient preference.

### 3.2. Pathologic Response and Surgical Outcomes

Among 184 surgically resected patients, the overall pCR rate (ypT0N0) was 19.3% (95% CI, 13.9–25.9).

Tumor regression grade (Dworak [28] TRG 0–4) distribution was as follows: TRG 0 (1.1%), TRG 1 (21.1%), TRG 2 (38.4%), TRG 3 (16.8%), and TRG 4 (19.5%). Major pathological response (MPR; TRG 3–4) was observed in 37.6% (N = 67) of resected patients. R0 resection (negative margins, ≥1 mm) was achieved in 94.5% (n = 173) of resected patients, R1 in 4.9% (n = 10), and R2 in one patient (0.5%). LVI was present in 23.2% (n = 42) and PNI in 15.9% (n = 29).

Univariable associations between baseline clinical/MRI features and major pathological response are presented in Table 2. These exploratory analyses evaluate whether MRI high-risk features (CRM involvement, EMVI, cT4, and cN2) and performance status are associated with achieving MPR after the sandwich TNT protocol and surgery.

### 3.3. Organ Preservation and Watch-and-Wait Outcomes

Of 205 patients treated with a sandwich TNT protocol, 21 (10.2%) were ultimately managed with the W&W strategy after restaging. W&W selection was restricted to patients with a clinical complete or near-complete response at the primary tumor site according to the predefined criteria. No patient underwent local excision for regrowth; therefore, radical-surgery-free survival was equivalent to TME-free survival in this cohort. W&W outcomes are detailed in Table 3. Among 21 patients selected for W&W, the median age was 63 years; 57.1% were male.

**Figure 2:**
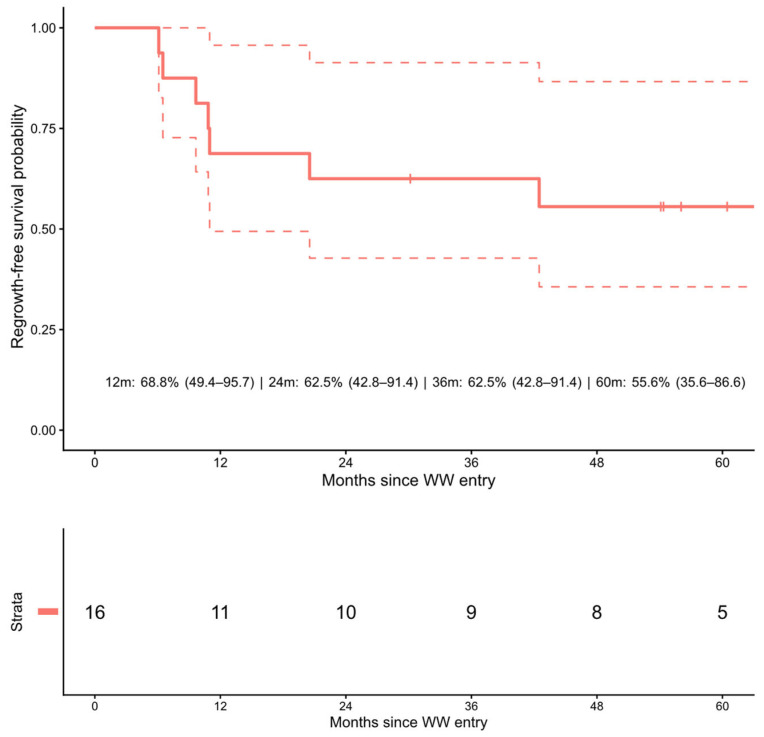
Regrowth-free survival among patients managed with the watch-and-wait (W&W) strategy after the sandwich total neoadjuvant therapy (TNT) protocol. The Kaplan–Meier curve with 95% confidence intervals (dotted lines) demonstrates regrowth-free survival probabilities of 68.8% (95% CI, 49.4–95.7) at 12 months, 62.5% (95% CI, 42.8–91.4) at 24 and 36 months, and 55.6% (95% CI, 35.6–86.6) at 60 months. The risk table below the curve shows the number at risk at each time point. Time zero is defined as entry into W&W protocol following clinical response assessment by MRI and endoscopy. W&W, watch-and-wait.

Over a median follow-up of 4.96 years from W&W protocol entry, local regrowth occurred in 7 (33.3%) (95% CI, 14.6–57.0%). Median time to regrowth was 10.8 months (range 6.1 months–42.5 months). Regrowth-free survival at 1/2/3/5 years was 68.8% (95% CI, 49.4–95.7), 62.5% (95% CI, 42.8–91.4), 62.5% (95% CI, 42.8–91.4), and 55.6% (95% CI, 35.6–86.6), respectively (Table 3 and Figure 2). Among patients with regrowth, 5 (71.4%) underwent salvage surgery; R0 was achieved in all patients.

### 3.4. Survival Outcomes

#### 3.4.1. Overall Survival

With a median follow-up of 5.04 years (95% CI, 4.86–5.43) from diagnosis, the observed follow-up time ranged from 0.71 to 9.01 years. There were 36 (17%) deaths among all 205 patients. Overall survival at 2, 3, and 5 years was 94.2% (95% CI, 91.0–97.4), 86.7% (95% CI, 82.0–91.6), and 81.1% (95% CI, 75.5–87.2), respectively (Table 4).

**Figure 3:**
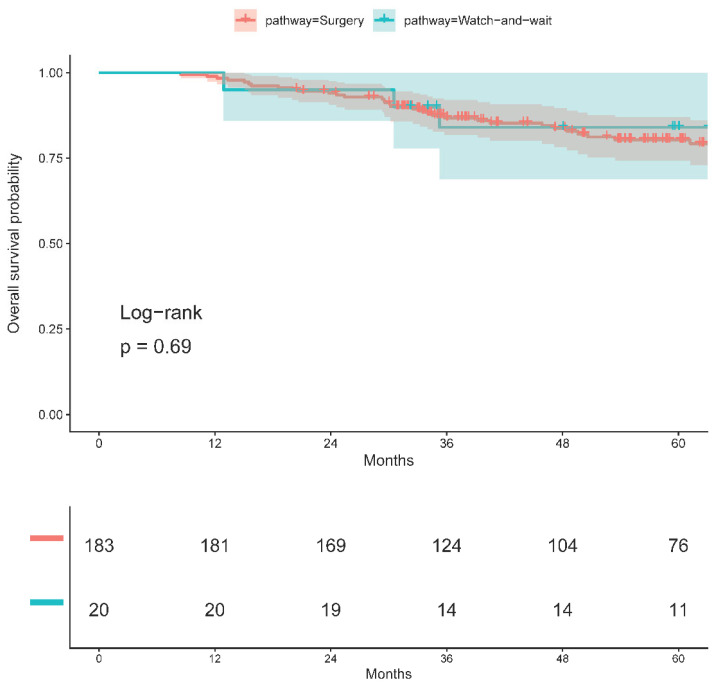
Overall survival (OS) by pathway. Kaplan–Meier curves comparing OS between watch-and-wait (W&W) and radical surgery (RS) cohorts following sandwich total neoadjuvant therapy (TNT) for locally advanced rectal cancer (LARC). The log-rank *p*-value shown.

When stratified by management pathway, OS at 2, 3, and 5 years in the resected cohort was 94.0% (95% CI, 90.6–97.5), 86.8% (95% CI, 81.8–92.0), and 80.3% (95% CI, 74.2–87.0). In the W&W cohort, corresponding OS estimates were 95.0% (95% CI, 85.9–100.0), 84.0% (95% CI, 68.8–100.0), and 84.0% (95% CI, 68.8–100.0) (Table 4). OS did not differ significantly between the resected and W&W pathways (log-rank *p* = 0.69, Figure 3).

#### 3.4.2. Disease-Free Survival

**Figure 4:**
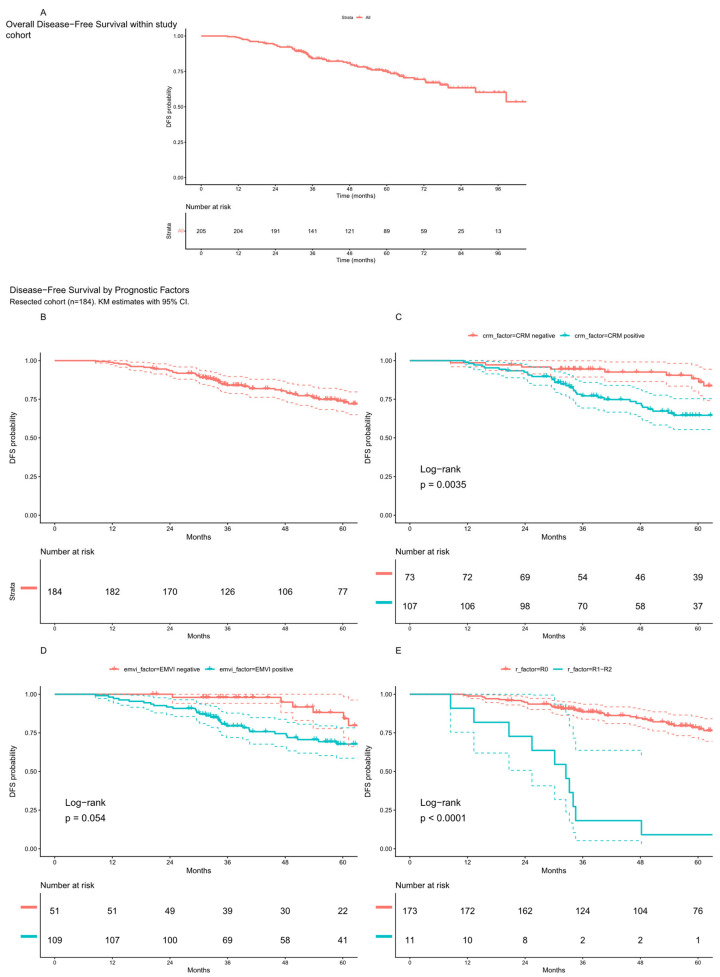
Overall disease-free survival (DFS) probability after sandwich TNT (**A**), DFS within resected patients (N = 184, (**B**)), and disease-free survival probability stratified by circumferential resection margin (CRM) status (**C**), extramural vascular invasion (EMVI) status (**D**), andby surgical margin (R0 vs. R1–R2 resection) status (**E**). The Kaplan-Meier curve are showed with 95% confidence intervals (dotted lines).

Among 205 patients, 56 experienced a DFS event. Median follow-up was 64.3 months (95% CI, 60.1–69.9). Estimated DFS was 93.7% (95% CI, 90.4–97.1) at 2 years, 84.1% (95% CI, 79.1–89.4) at 3 years, and 75.2% (95% CI, 69.0–82.0) at 5 years (Figure 4, Panel A).

Among 184 resected patients, disease-free survival at 2, 3, and 5 years was 93.5% (95% CI, 90–97.1), 83.5% (95% CI, 78.1–89.2), and 73.5% (95% CI, 66.7–80.9), respectively, with the median not reached. Figure 4 (Panel B) depicts the overall Kaplan–Meier DFS probability curve for these patients. Figure 4 illustrates DFS stratified by CRM status (Panel C), EMVI status (Panel D), and surgical margin status (Panel E). CRM-positive patients exhibited inferior 3-year DFS compared to CRM-negative patients (77% vs. 94%; 70 vs. 54 at risk; log-rank *p* = 0.003). EMVI-positive status was associated with worse 3-year DFS (79% vs. 98%; 69 vs. 39 at risk; *p* < 0.001). Surgical margin status showed the strongest prognostic impact, with R0 resections achieving 89% 3-year DFS compared to 18% for R1–R2 resections (124 vs. 2 at risk; *p* < 0.001).

**Figure 5:**
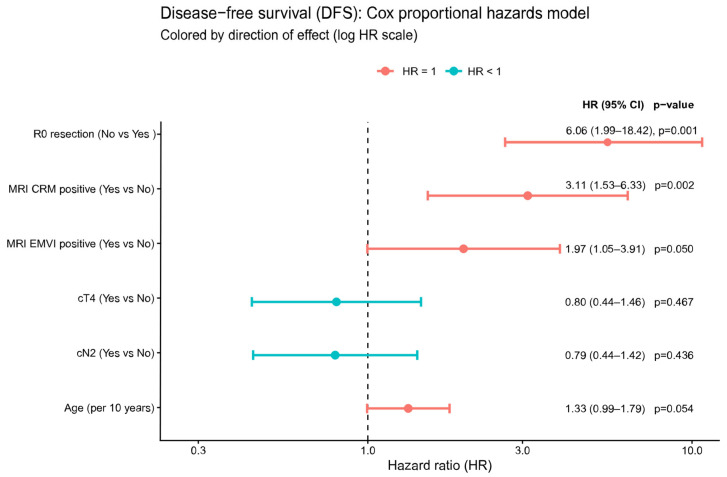
Multivariable Cox model for disease-free survival (DFS) in resected patients. Forest plot displaying adjusted hazard ratios (HRs) with 95% confidence intervals for MRI-based and pathological prognostic factors, plotted on a logarithmic scale. A vertical dashed line indicates the null value (HR = 1.0). Predictors to the right of the line (HR > 1) are associated with higher risk of DFS events, whereas those to the left (HR < 1) are associated with lower risk. The model concordance index was 0.723 (95% CI, 0.652–0.794), with significant global likelihood ratio, Wald, and score tests (all *p* ≤ 0.003). DFS, disease-free survival; HR, hazard ratio; CI, confidence interval; CRM, circumferential resection margin; EMVI, extramural vascular invasion; cT4, clinical T4 stage (tumor invades other organs/structures per AJCC staging); cN2, clinical N2 stage (≥4 regional lymph node metastases per AJCC staging); R0, microscopically margin-negative resection.

In multivariable Cox regression analysis (Figure 5), R0 resection was associated with a markedly lower risk of DFS events (R1/2 vs. R0 HR 6.06, 95% CI, 1.99–18.42, *p* = 0.001), consistent with the strong adverse effect of any margin involvement. MRI CRM positivity independently worsened DFS (HR 3.11, 95% CI, 1.53–6.33, *p* = 0.002), and MRI EMVI positivity showed a significant association with poorer DFS (HR 1.97, 95% CI, 1.05–3.91, *p* = 0.050). Age showed a borderline effect, with a 33% relative increase in hazard per 10-year increment (HR 1.33, 95% CI, 0.99–1.79, *p* = 0.054). Clinical cT4 and cN2 stages did not significantly influence DFS after adjustment (cT4: HR 0.80, 95% CI, 0.44–1.46, *p* = 0.467; cN2: HR 0.79, 95% CI, 0.44–1.42, *p* = 0.436).

#### 3.4.3. Local Recurrence

Local recurrence is reported as an incidence proportion with exact 95% confidence intervals and as a landmark incidence among patients with at least 2, 3, and 5 years of follow-up. Local recurrence occurred in 17 (9.3%) of resected patients. Among patients with ≥24, ≥36, and ≥60 months follow-up, local recurrence rates were 8.3%, 4%, and 2.7%, respectively.

Incidence appeared higher in patients with CRM+, EMVI+, cT4, or cN2 disease, consistent with baseline risk features.

#### 3.4.4. Exploratory Comparison of Complete Responders—Surgical pCR Versus cCR Under Watch-and-Wait

Among patients achieving complete response, 35 had a pathological complete response (pCR, ypT0N0) after surgery, and 21 were managed non-operatively with W&W following a cCR at restaging. In the W&W cohort, 7 of 21 patients (33.3%) developed local regrowth at the primary site, 5 of whom (71.4%) underwent salvage TME with R0 resection in all cases. No subsequent post-resection pelvic recurrences occurred in this subset. All pelvic local recurrences in the overall cohort (17/184 resected patients, 9.3%) arose in patients without a complete response.

**Figure 6:**
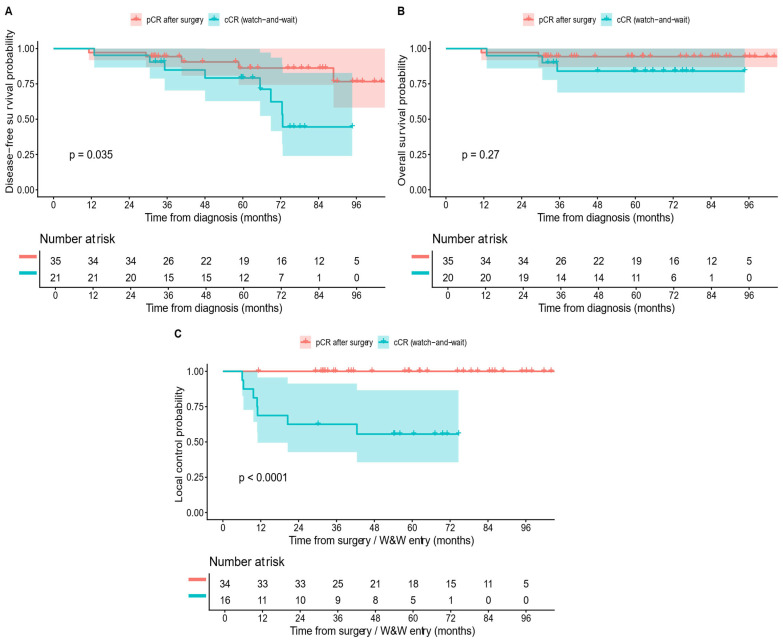
Exploratory comparison of complete responders: surgical pathological complete response (pCR) and clinical complete response (cCR) managed with watch-and-wait (W&W). (**A**) Disease-free survival (DFS) from diagnosis among complete responders, comparing patients with pCR treated with radical surgery versus those with cCR managed with watch-and-wait. (**B**) Overall survival (OS) from diagnosis in the same subgroups. (**C**) Cumulative incidence of local failure, showing absence of post-resection local recurrence in pCR/surgery patients and the pattern of local regrowth in cCR/W&W patients. Salvageable local regrowth was analyzed separately from post-resection local recurrence and does not reflect incurable pelvic failure. Pathway-specific survival estimates are reported descriptively; direct comparisons between surgery and W&W are exploratory and limited by non-randomized, response-dependent allocation. Abbreviations: pCR, pathological complete response; cCR, clinical complete response; W&W, watch-and-wait.

DFS from diagnosis was significantly higher in the pCR/surgery group compared with the cCR/W&W group (Figure 6A; log-rank *p* = 0.035). Three-year DFS was 94.3% (95% CI, 86.9–100) in the pCR group and 84.8% (95% CI, 70.3–100) in the cCR/W&W group. In contrast, OS did not differ significantly between the two strategies, with high long-term survival observed in both cohorts (Figure 6B; log-rank *p* = 0.27). At 5 years, OS was 94.3% (95% CI, 86.9–100) in the pCR group and 84% (95% CI, 68.8–100) in the cCR/W&W group. Differences were most pronounced for local control. No post-resection pelvic recurrences were observed among patients with pCR after surgery, whereas patients managed with W&W experienced local regrowth events (Figure 6C, log-rank *p* < 0.0001). Because these groups were not randomized, the *p*-values are provided for descriptive completeness and should not be interpreted as comparative effectiveness evidence.

## 4. Discussion

To the best of our knowledge, this is the first study reporting long-term results of sandwich TNT. Only one retrospective international study recently reported 5-year results of different TNT approaches outside clinical trials on 1585 patients, in which sandwich TNT was used in only 17% of them [31]. This retrospective study of a consecutive cohort of 205 patients with LARC treated uniformly with a sandwich TNT strategy provides real-world implementation data for this strategy in a tertiary referral setting enriched for MRI-defined high-risk disease. Across the entire cohort, the sandwich TNT approach yielded an overall complete response rate of 29.5% (pCR 19.3%, cCR 10.2%), a major pathological response in 37.6%, and R0 resection in 94.5% of operated patients. Five-year OS and DFS for the whole TNT cohort were 81.1% (95% CI, 75.5–87.%) and 75.2% (95% CI, 69.0–82.%), respectively, and post-resection local recurrence remained below 10%. These whole-cohort endpoints are clinically informative because they reflect the composite effectiveness of a protocol that includes both radical surgery and a structured watch-and-wait (W&W) pathway, mirroring contemporary practice where organ preservation is increasingly integrated into TNT-based management [32]. The reported results should be interpreted as observational evidence describing feasibility and outcomes of one institutional platform, not as proof that sandwich TNT is superior to better-studied induction-only or consolidation-only TNT sequences.

Our study results demonstrate substantial pathological stage migration, acceptable long-term oncologic outcomes, and the feasibility of selective W&W management. At the same time, the cohort’s high-risk MRI features, non-random pathway allocation, and lack of prospectively collected toxicity and quality-of-life data limit the strength of comparative inference.

### 4.1. Tumor Response, Stage Migration and Survival in a High-Risk Cohort

When interpreted in isolation, a pCR rate around 19% may appear modest relative to some contemporary TNT trials such as RAPIDO and PRODIGE–23, in which intensified neoadjuvant strategies produced higher pCR rates in broadly selected stage II–III populations [7,9,12]. However, direct numerical comparison is problematic for several reasons. First, the baseline risk profile in the present cohort is considerably more adverse than in most published randomized series: 61% had an MRI-threatened or involved circumferential resection margin (CRM), 66% were EMVI-positive, 60% had cN2 nodal disease, and 41% had cT4 disease. Second, the present study was retrospective and included a W&W pathway, so the overall cohort cannot be benchmarked against surgery-only randomized trial populations by simply juxtaposing percentages. Third, pCR in this study was calculated among evaluable resected patients, whereas W&W entry after cCR/near-cCR was a separate organ-preservation outcome. Nonetheless, the long-term survival estimates observed here suggest that meaningful disease control can be achieved in routine practice despite a notably adverse baseline case-mix. This is clinically relevant because tertiary referral cohorts often include precisely those MRI-defined high-risk patients for whom robust non-trial data are sparse.

MRI-defined CRM involvement and EMVI are well-established adverse prognostic features and can outperform TNM staging in predicting oncologic outcomes, supporting the clinical relevance of reporting baseline MRI risk composition when benchmarking TNT results. In this context, the observed proportion of patients downstaged to ypT0–2 and/or ypN0, major pathological response rate (Dworak TRG 3–4; 37.6%), and high R0 resection rate (94.5%) indicate clinically meaningful tumor regression in an exceptionally high-risk population.

Importantly, the whole-cohort 5-year DFS of 75.2% and OS of 81.1% are comparable to long-term TNT trial benchmarks, suggesting that the sandwich sequencing used here can deliver durable disease control in high-risk routine practice. This is aligned with the broader TNT evidence base demonstrating improvements in systemic control and DFS compared with conventional approaches, even though trial populations and treatment platforms differ.

Large phase III TNT trials such as RAPIDO and PRODIGE–23 have demonstrated improvements in distant metastasis and DFS compared with conventional CRT followed by selective adjuvant chemotherapy, but in cohorts with somewhat different case-mix and staging criteria [7,9,12]. In PRODIGE–23, induction mFOLFIRINOX followed by CRT and surgery yielded a pCR rate of 27.5%, but enrolled patients with cT3–4 M0 disease and lower MRI-defined risk than in the present series [11,12]. RAPIDO, which used short-course radiotherapy followed by consolidation chemotherapy before TME, reported a substantial increase in pCR and a reduction in distant metastases at the cost of a higher locoregional recurrence risk (LRR) in long-term follow-up [9]. A recent subanalysis attributed this excess LRR to patients undergoing sphincter-preserving surgery with a distal resection margin ≤ 10 mm, where the 8-year LRR rate after TNT was 25.4% versus 1.8% after CRT (HR 15.51) [33]. Our radiotherapy platform differs from the short-course regimen used in RAPIDO. This finding emphasizes that local-control trade-offs are not generic to all TNT approaches but may be relevant when marked tumor shrinkage misguides surgical margin planning, supporting caution in any indirect comparison with our IMRT-SIB-based program.

Within this broader TNT landscape, our findings support the feasibility of sandwich TNT in a high-risk LARC cohort, but they do not establish a preference for this sequence over other TNT approaches.

### 4.2. Persistent Prognostic Impact of MRI-Defined Anatomy and Margins

Despite the overall efficacy of TNT in inducing tumor regression, our data highlight that pretreatment MRI risk factors and surgical margin status remain critical prognostic determinants. Patients whose tumors threatened or breached the mesorectal fascia on MRI had significantly worse outcomes, even after TNT and surgery. In our multivariable Cox analysis, an MRI-positive CRM was associated with more than a tripling of the hazard of relapse (HR 3.11, 95% CI, 1.53–6.33). Likewise, EMVI positivity was associated with a near-doubling of relapse risk (HR 1.97, 95% CI, 1.05–3.91).

Perhaps most striking is the influence of surgical margin quality. Patients undergoing an R1/R2 resection in our cohort had markedly inferior DFS compared to those with R0 resection (3-year DFS 18% vs. 89%), corresponding to a 6-fold increased hazard of recurrence for incomplete resections (HR 6.06; 95% CI, 1.99–18.42). All 36 recorded deaths in our study occurred in patients with R1/R2 or other high-risk features, underscoring that clear margins (R0) are imperative for cure. These results emphasize that even in the TNT era, precise MRI staging and high-quality surgery are essential. We advocate upfront MRI risk stratification (e.g., for CRM, EMVI, and tumor deposits) to guide therapy intensity and surgical planning. Patients with threatened CRM or bulky disease should be referred to experienced surgeons, as achieving an R0 resection can dramatically improve outcomes (DFS ~ 73% at 5 years in our R0 patients). In summary, TNT downstages disease but does not abrogate the prognostic importance of baseline risk factors or surgical technique. Ongoing refinements in MRI (e.g., tumor regression grading on restaging MRI) and incorporation of biomarkers may further inform which patients need augmented therapy beyond standard TNT.

### 4.3. Sandwich TNT Protocol in Contemporary Sequencing Debates

The optimal sequencing of TNT for LARC remains an area of active investigation. Contemporary randomized trials have primarily compared induction chemotherapy followed by CRT with CRT followed by consolidation chemotherapy, rather than more complex schedules. Overall, TNT has improved pathological response and reduced distant failure compared with conventional long-course CRT with selective adjuvant chemotherapy, as shown in PRODIGE–23 [12] and related studies, and is now recommended by the European Society for Medical Oncology and other international guidelines for MRI-defined high-risk stage II–III rectal cancer [34]. The CAO/ARO/AIO–12 and OPRA trials further suggest that CRT followed by consolidation chemotherapy may improve pCR and sustained cCR/organ preservation, without compromising disease-free survival, and is therefore preferred in many centers when organ preservation is a priority [10,17,35].

A distinctive component of our cohort is the radiotherapy delivery regimen, consisting of long-course hypofractionated IMRT-SIB delivering 41.8 Gy to the elective pelvic volume with a boost to 46.2 Gy for cT3 and 48.4 Gy for cT4 tumors in 22 fractions with concurrent capecitabine. The institutional sandwich TNT pathway was developed stepwise from prior prospective institutional experience, including a phase II study of induction chemotherapy, chemoradiotherapy, and consolidation chemotherapy and a phase II study establishing the feasibility of IMRT-SIB chemoradiotherapy [21,36]. This schedule differs from conventional long-course CRT fractionation commonly used across major TNT trials. By extending IMRT-SIB–based chemoradiotherapy into a large real-world sandwich TNT cohort with long follow-up, the present study helps address a practical evidence gap, namely whether this non-standard fractionation can achieve outcomes comparable to established neoadjuvant CRT schedules when embedded within modern TNT. This hybrid approach, delivered uniformly to all patients, attempts to capture the benefits of both sequences: early systemic therapy to tackle micrometastases and additional chemotherapy after radiation to maximize tumor regression prior to surgery. Phase II studies of similar sandwich regimens have reported pCR rates around 30–40% [37], exceeding those of induction regimens, although randomized evidence is sparse. Notably, delivering chemotherapy both before and after CRT did not appear to compromise tolerability in our series—90% of patients completed all planned cycles.

While direct comparisons between sequencing strategies await prospective trials, our real-world data suggest that a sandwich approach can achieve oncologic outcomes on par with induction-only or consolidation-only TNT. This may be particularly relevant for centers or patients aiming to balance maximal tumor response with early systemic therapy. Moving forward, randomized studies (or pooled analyses) formally comparing mixed-sequence TNT to single-sequence regimens would help determine if outcomes can be further optimized by such adaptive scheduling. Potential theoretical advantages of our institutional sandwich TNT protocol include earlier treatment of micrometastatic disease, additional time for response maturation before surgery, and flexibility to combine an established long-course local platform with pre- and post-radiotherapy systemic therapy. Potential disadvantages must also be acknowledged, such as greater logistical complexity, cumulative toxicity risk, possible overtreatment of some patients, and heterogeneity in systemic therapy delivery over time. Because detailed prospective toxicity and functional data were not available in this retrospective cohort, these trade-offs cannot be fully quantified here.

### 4.4. Watch-and-Wait Outcomes and Salvageability

Within the whole TNT cohort, 10.2% of patients were managed by W&W after achieving cCR, which represents a pragmatic organ-preservation rate in a cohort dominated by MRI high-risk features.

Two-thirds of our W&W patients were able to avoid surgery long-term, and salvage TME was successful in 100% of those who did recur. This is consistent with the OPRA trial and IWWD registry, which reported high salvage rates and no survival penalty for W&W [18,38]. Thus, for patients desiring organ preservation and meeting strict response criteria, W&W is a reasonable option. It must be coupled with rigorous surveillance. Patients should also be counseled that in real-world settings, up to 40–50% may ultimately require surgery due to tumor regrowth or indeterminate findings.

Taken together, our W&W results support feasibility after sandwich TNT, while the regrowth rate underscores the need for strict selection criteria and intensive surveillance, particularly in MRI high-risk patients. W&W should be considered mainly for patients in whom organ preservation is clinically meaningful and who demonstrate a robust or maturing complete response on multimodal restaging, can adhere reliably to repeated MRI/endoscopic surveillance, and have immediate access to salvage surgery if regrowth occurs.

### 4.5. Limitations and Strengths

This study has several limitations. It is a single-institution retrospective analysis, which carries inherent risks of selection bias and unmeasured confounding. Further on, the choice of W&W was not randomized and was influenced by post-treatment response assessment and multidisciplinary judgement, which could bias comparisons. The sample size of the W&W cohort (n = 21) was relatively small, limiting statistical power to detect minor differences and to perform extensive subgroup analyses (for instance, outcomes by cCR stringency or salvage timing). Additionally, pathologic tumor regression grading was performed by institutional pathologists without central review; while all used a standard Dworak TRG scheme, some inter-observer variability is possible. We also note that our median follow-up (~5 years for surviving patients) may be insufficient to capture very late recurrences, though >90% of relapses in rectal cancer occur within 5 years. Acute toxicity, late toxicity, bowel function, sexual and urinary function, and patient-reported quality of life were not systematically collected and therefore cannot be evaluated. Microsatellite status was not uniformly available across the full study period, and standardized grading of TME specimen quality was not consistently documented, limiting additional biological and surgical quality analyses. Despite these limitations, the study’s strengths include a consecutive real-world cohort reflecting routine practice (enhancing generalizability), comprehensive MRI staging data for all patients, and mature survival follow-up exceeding 5 years. Moreover, the uniformity of the TNT protocol and surgical technique at our center provides a consistent treatment backdrop against which outcomes were assessed.

### 4.6. Clinical Implications and Future Directions

The findings from this high-risk real-world cohort underscore the viability and effectiveness of sandwich TNT, integrating both induction and consolidation chemotherapy, in achieving favorable long-term oncologic outcomes. MRI-based risk stratification (CRM, EMVI) should guide treatment intensification and patient selection, as baseline tumor location and morphologic features remain independent prognostic factors even after TNT [14,16]. W&W with rigorous surveillance is a safe, effective organ-preservation strategy when structured MRI/endoscopy surveillance is available, multidisciplinary assessment confirms adequate cCR/near-cCR, patient commitment to surveillance is assured, and timely access to salvage surgery is guaranteed. However, patients must be counseled that regrowth occurs in 40–50% of W&W candidates in pragmatic settings, and careful baseline risk factor assessment is essential [10,17,18]. Resection margin status (R0 vs. R1–R2) is the dominant modifiable prognostic factor and should motivate specialized surgical referral and TME quality focus, particularly for high-risk disease (CRM+, EMVI+, and cT4) [39,40,41]. Chemotherapy dose intensity (RDI > 85%) should be prioritized through proactive toxicity management and supportive care, as supported by literature on improved survival [42,43]. Regarding selection, sandwich TNT is best suited for patients with MRI-defined high-risk features (CRM+, EMVI+, cT4, and cN2) who can tolerate multi-agent chemotherapy and for whom maximizing tumor response before surgery is prioritized. W&W should be reserved for strictly defined cCR/near-cCR on both MRI and endoscopy, with patient commitment to intensive surveillance (especially in the first 2–3 years), access to an experienced multidisciplinary team, and guaranteed timely salvage surgery.

It must be acknowledged that sandwich TNT has not been directly compared with induction-only or consolidation-only TNT in randomized trials. In the absence of prospective comparative data, the choice of sequencing should be individualized based on patient factors, tumor characteristics, and institutional expertise rather than assuming the superiority of any single strategy.

## 5. Conclusions

This single-institution real-world cohort suggests that sandwich TNT with an IMRT-SIB backbone is a feasible option for MRI-defined high-risk LARC and can achieve meaningful tumor regression, high R0 resection rates, and durable survival. Structured W&W enabled organ preservation, with a 5-year TME-free survival of 73.1%, but regrowth in one-third of cases confirms the need for strict response criteria, intensive surveillance, and prompt salvage capability. Baseline MRI risk features and resection margin status remained key determinants of outcome, underscoring the continued importance of expert staging and high-quality surgery in the TNT era.

## Figures and Tables

**Table 1:** Clinicopathological characteristics of the study cohort.

Characteristic	Overall (n = 205) ^1^	Surgery (n = 184) ^1^	Watch-and-Wait (n = 21) ^1^
**Sex**			
Male	133 (65%)	121 (66%)	12 (57%)
Female	72 (35%)	63 (34%)	9 (43%)
**Age at diagnosis (years)**			
Median (IQR)	61 (52, 67)	61 (52, 67)	63 (55, 68)
Min–Max	32–80	32–80	33–77
**ECOG performance status**			
0	151 (74%)	135 (73%)	16 (76%)
1	53 (26%)	48 (26%)	5 (24%)
≥2	1 (0.5%)	1 (0.5%)	0 (0%)
**Baseline CEA (ng/mL)**			
Median (IQR)	4 (2, 11)	4 (2, 11)	4 (2, 8)
Min–Max	0–375	0–375	1–23
**Tumor grade**			
G1 (Well)	26 (15%)	26 (17%)	0 (0%)
G2 (Moderate)	126 (73%)	106 (69%)	20 (100%)
G3 (Poor)	13 (7.5%)	13 (8.5%)	0 (0%)
Mucinous/Undifferentiated	8 (4.6%)	8 (5.2%)	0 (0%)
Unknown	32	31	1
**Distance from anal verge**			
0–5 cm	84 (41%)	75 (41%)	9 (43%)
5.1–10 cm	94 (46%)	84 (46%)	10 (48%)
10.1–15 cm	27 (13%)	25 (14%)	2 (9.5%)
**Clinical T stage (cT)**			
2	5 (2.4%)	5 (2.7%)	0 (0%)
3	116 (57%)	98 (53%)	18 (86%)
4	84 (41%)	81 (44%)	3 (14%)
**Clinical N stage (cN)**			
0	14 (6.8%)	10 (5.4%)	4 (19%)
1	67 (33%)	58 (32%)	9 (43%)
2	124 (60%)	116 (63%)	8 (38%)
**MRI CRM threatened/involved**	122 (61%)	107 (59%)	15 (71%)
Missing	4	4	0
**MRI EMVI positive**	120 (66%)	110 (68%)	10 (48%)
Missing	23	23	0

^1^ n (%). Abbreviations: CEA, carcinoembryonic antigen; cN, clinical nodal stage; cT, clinical T stage; CRM, circumferential resection margin (threatened/involved on baseline MRI); ECOG, Eastern Cooperative Oncology Group performance status; EMVI, extramural vascular invasion (positive on baseline MRI); IQR, interquartile range; MRI, magnetic resonance imaging.

**Table 2:** Univariable predictors of major pathological response (MPR = TRG 3–4).

	Descriptive			Logistic Regression			
Characteristic	Overall n = 178 ^1^	TRG 1–2 n = 111 ^1^	TRG 3–4 n = 67 ^1^	n	OR	95% CI	*p*-Value
**Sex**				178			
Male	117 (66%)	72 (65%)	45 (67%)		-	-	
Female	61 (34%)	39 (35%)	22 (33%)		0.90	0.47–1.71	0.8
**ECOG PS**				178			
0	131 (74%)	79 (71%)	52 (78%)		-	-	
1	46 (26%)	31 (28%)	15 (22%)		0.74	0.35–1.48	0.4
≥2	1 (0.6%)	1 (0.9%)	0 (0%)		NE		>0.9
**Histologic grade**				147			
G1 (Well)	24 (16%)	12 (13%)	12 (24%)		-	-	
G2 (Moderate)	103 (70%)	69 (72%)	34 (67%)		0.49	0.20–1.22	0.12
G3 (Poor)	12 (8.2%)	8 (8.3%)	4 (7.8%)		0.50	0.11–2.05	0.3
Mucinous/Undifferentiated	8 (5.4%)	7 (7.3%)	1 (2.0%)		0.14	0.01–0.98	0.089
**CEA (pre)**	4 (2–11)	5 (2–11)	4 (2–9)	178	1.00	0.99–1.01	> 0.9
**Tumor height**				178			
0–5 cm	74 (42%)	47 (42%)	27 (40%)		-	-	
5.1–10 cm	80 (45%)	47 (42%)	33 (49%)		1.22	0.64–2.35	0.5
10.1–15 cm	24 (13%)	17 (15%)	7 (10%)		0.72	0.25–1.89	0.5
**cT stage**				178			
2	4 (2.2%)	2 (1.8%)	2 (3.0%)		-	-	
3	97 (54%)	59 (53%)	38 (57%)		0.64	0.07–5.55	0.7
4	77 (43%)	50 (45%)	27 (40%)		0.54	0.06–4.70	0.5
**cN stage**				178			
0	9 (5.1%)	6 (5.4%)	3 (4.5%)		-	-	
1	55 (31%)	41 (37%)	14 (21%)		0.68	0.16–3.57	0.6
2	114 (64%)	64 (58%)	50 (75%)		1.56	0.39–7.69	0.5
**CRM+**				178			
No	71 (39%)	42 (39%)	28 (42%)		-	-	
Yes	104 (58%)	66 (59%)	38 (57%)		0.89	0.48–1.66	0.7
**EMVI+**				178			
No	50 (28%)	31 (31%)	19 (34%)		-	-	
Yes	105 (59%)	68 (61%)	37 (55%)		0.78	0.42–1.44	0.4
**Dose reduction**				178			
No reduction	165 (93%)	102 (92%)	63 (94%)		-	-	
Oxaliplatin reduced	6 (3.4%)	3 (2.7%)	3 (4.5%)		1.62	0.29–8.98	0.6
Capecitabine reduced	3 (1.7%)	3 (2.7%)	0 (0%)		NE		>0.9
Both reduced	4 (2.2%)	3 (2.7%)	1 (1.5%)		0.54	0.03–4.32	0.6

^1^ n (%); median (Q1–Q3); abbreviations: CI, confidence interval; OR, odds ratio; MPR, major pathologic response (TRG 3–4 Dworak tumor regression grade [28]); NE, not estimable.

**Table 3:** Watch-and-wait (W&W) management outcomes. Clinical characteristics and oncologic results for 21 patients selected for W&W following total neoadjuvant therapy (TNT).

Metric	Value
W&W cohort size	21
Median age (IQR), years	63 (55–68)
Male sex, n (%)	12/21 (57.1%)
Median follow-up from W&W entry (IQR), years	4.96 (2.50–5.57)
Local regrowth, n/N (%)	7/21 (33.3%)
Local regrowth, 95% CI	14.6–57.0%
Median time to regrowth (range), months	10.8 (6.1–42.5)
Regrowth-free survival at 1/2/3/5 years (95% CI)	1-year: 68.8% (49.4–95.7); 2-year: 62.5% (42.8–91.4); 3-year: 62.5% (42.8–91.4); 5-year: 55.6% (35.6–86.6)
Salvage (TME) surgery among regrowth, n/N (%)	5/7 (71.4%)
R0 among salvage resections, n/N (%)	5/5 (100.0%)
TME-free survival at 5 years (95% CI)	73.1% (55.0–97.2)

Abbreviations: W&W, watch-and-wait; IQR, interquartile range; CI, confidence interval; R0, microscopically margin-negative resection; TME, total mesorectal excision.

**Table 4:** Overall survival estimation at prespecified time points by treatment pathway.

	Overall Survival Estimation at Prespecified Time Points (%, 95% CI)		
	Month 24	Month 36	Month 60
**Whole cohort**	94.2% (91.0–97.4)	86.7% (82.0–91.6)	81.1% (75.5–87.2)
**Surgery**	94.0% (90.6–97.5)	86.8% (81.8–92.0)	80.3% (74.2–87.0)
**W&W**	95.0% (85.9–100.0)	84.0% (68.8–100.0)	84.0% (68.8–100.0)

W&W, watch-and-wait.

## Data Availability

Data is contained within the manuscript. Further information about the data may be provided on request.

## Abbreviations

The following abbreviations are used in this manuscript:

AJCC	American Joint Committee on Cancer
CAO-ARO-AIO	German Rectal Cancer Study Group acronym used in trial name (CAO/ARO/AIO-12)
CAPOX	Capecitabine plus oxaliplatin
CEA	Carcinoembryonic antigen
CI	Confidence interval
cCR	Clinical complete response
cM/cM+	Clinical M stage/clinical M1 (synchronous metastatic disease)
cN	Clinical nodal stage (TNM)
cT	Clinical tumor stage (TNM)
CR	Complete response
CRM	Circumferential resection margin
CRT	Chemoradiotherapy
DFS	Disease-free survival
DRE	Digital rectal examination
ECOG PS	Eastern Cooperative Oncology Group performance status
EMVI	Extramural vascular/venous invasion
EORTC	European Organisation for Research and Treatment of Cancer
ERID-KSOPKR	Institutional Review Board identifier (as written)
ERIDEK	Institutional Ethical Committee identifier (as written)
ESMO	European Society for Medical Oncology
FOLFIRINOX	Fluorouracil, leucovorin, irinotecan, and oxaliplatin combination regimen
FOLFOX6/mFOLFOX6	(Modified) FOLFOX6 regimen (as cited)
HR	Hazard ratio
ICRU	International Commission on Radiation Units and Measurements
IMRT	Intensity-modulated radiation therapy
IMRT-SIB	Intensity-modulated radiation therapy with simultaneous integrated boost
IQR	Interquartile range
IWWD	International Watch & Wait Database
LARC	Locally advanced rectal cancer
LN	Lymph node
LR	Local recurrence
LVI	Lymphovascular invasion
mFOLFIRINOX	Modified FOLFIRINOX
MPR	Major pathological response
MRI	Magnetic resonance imaging
mrEMVI	MRI-detected extramural vascular invasion
MSI	Microsatellite instability
OPRA	Organ Preservation in Rectal Adenocarcinoma trial acronym
OR	Odds ratio
OS	Overall survival
pCR	Pathological complete response
PNI	Perineural invasion
PRODIGE	French cooperative group acronym in trial name (PRODIGE-23)
R0/R1/R2	Resection margin status: negative/microscopic positive/macroscopic positive
RAPIDO	RAPIDO trial acronym
RDI	Relative dose intensity
RS	Radical surgery
SCRT	Short-course radiotherapy
SIB	Simultaneous integrated boost
STROBE	Strengthening the Reporting of Observational Studies in Epidemiology
TD	Tumor deposits
TME	Total mesorectal excision
TNM	Tumor, nodes, and metastases classification system
TNT	Total neoadjuvant therapy
TRG	Tumor regression grade
W&W	Watch-and-wait
ypT0N0	Pathological stage T0N0 after neoadjuvant therapy
95% CI	95% Confidence Interval
